# Altered glymphatic function in nasopharyngeal carcinoma following radiotherapy: novel insights from choroid plexus volume and free-water fraction analyses

**DOI:** 10.3389/fnins.2026.1690724

**Published:** 2026-05-04

**Authors:** Lingling Deng, Jianchun Peng, Keyang Zhou, Kun Fan, Yuting Xia, Shuping Zhang, Li Li, Jian-ming Gao, Na Jiang, Youming Zhang

**Affiliations:** 1 Department of Radiology, The Second Affiliated Hospital, University of South China, Hengyang, Hunan, China; 2 Department of Radiology, Xiangya Hospital, Central South University, Changsha, Hunan, China; 3 National Clinical Research Center for Geriatric Diseases, Xiangya Hospital, Central South University, Changsha, Hunan, China; 4 Department of Ultrasound Imaging, The Second People's Hospital of Yichun City, Yichun, Jiangxi, China; 5 Sun Yat-sen University Cancer Center, State Key Laboratory of Oncology in South China, Collaborative Innovation Center for Cancer Medicine, Guangzhou, China; 6 Department of Radiation Oncology, Collaborative Innovation Center for Cancer Medicine, State Key Laboratory of Oncology in South China, Sun Yat-sen University Cancer Center, Guangzhou, China; 7 Department of Radiology and Nuclear Medicine, The Fifth Affiliated Hospital of Jinan University (Shenhe People’s Hospital), Heyuan, Guangdong, China

**Keywords:** choroid plexus, free-water fraction, glymphatic dysfunction, nasopharyngeal carcinoma, radiotherapy

## Abstract

**Background and purpose:**

Radiotherapy (RT) often causes delayed radiation-induced brain injury (RBI) with unclear pathophysiology; emerging evidence links this to glymphatic dysfunction, but radiation effects on cerebrospinal fluid (CSF) dynamics and interstitial fluid-CSF exchange are unstudied. Thus, we used choroid plexus (CP) volume and free-water fraction (FWF) imaging to assess glymphatic changes in Nasopharyngeal carcinoma (NPC) patients after RT.

**Materials and methods:**

In this cross-sectional cohort of 101 NPC patients (45 pre-RT and 56 post-RT) underwent 3 T MRI, including T1-weighted and diffusion tensor imaging. Automated CP segmentation and tract-specific FWF analysis are performed. Spearman correlation models assessed radiation-dose relationships with CP volume and Whiter matter (WM) FWF.

**Results:**

We observed that post-RT patients exhibited significant bilateral CP enlargement (total CP: 2560.56 ± 636.72 mm^3^, left: 1196.92 ± 334.53 mm^3^, right: 1363.64 ± 365.84 mm^3^; all *p* < 0.05) and elevated FWF in critical WM tracts, including the pontine crossing tract (PCT), bilateral corticospinal tracts (CST), middle cerebellar peduncle, right inferior cerebellar peduncle, and left medial lemniscus. Radiational dose exhibit strong dose-dependent correlations with CP volume and WM FWF. Maximum doses to the brainstem (MDRT_BS) and left temporal lobe (MDRT_LT) showed the strongest associations: MDRT_LT correlated with left CP volume (*r* = 0.599, *p* < 0.001), right CP volume (*r* = 0.585, p < 0.001), and bilateral CST FWF (left: *r* = 0.414, *p* = 0.005; right: *r* = 0.354, *p* = 0.017). CP volume positively correlated with FWF in the PCT and CST (left CST vs. total CP: *r* = 0.374, *p* = 0.011). These associations remained significant after adjusting for age, gender, and intracranial volume (*r* = 0.31–0.58, all *p* < 0.05).

**Conclusion:**

The observed association between choroid plexus enlargement and elevated white matter free-water fraction suggests RT-associated glymphatic dysfunction in NPC, offers a novel perspective on the pathogenesis of RBI.

## 1 Introduction

Nasopharyngeal carcinoma (NPC), a malignant epithelial neoplasm arising from the mucosal lining of the nasopharynx, necessitates radiotherapy (RT) as its primary treatment modality due to the tumor’s complex anatomical location and high radiosensitivity (Chen et al., 2019). Although RT has markedly improved disease control and survival (Chua et al., 2016), it is associated with delayed complications. Among these, radiation-induced brain injury (RBI) has emerged as a major clinical concern. RBI typically manifests months to years after RT, presenting as progressive neuropsychiatric deficits such as cognitive impairment and epileptic seizures (Chen et al., 2019). Despite its clinical significance, the exact pathophysiology of RBI remains understood, impeding the development of targeted preventive strategies.

The pathogenesis of RBI involves a cascade of events including direct DNA damage, oxidative stress, neuroinflammation, and microvascular compromise, ultimately leading to cortical atrophy, white matter (WM) demyelination, and microstructural degradation (Zheng et al., 2022). Preclinical evidence also indicates that radiation-induced inflammatory mediators (e.g., IL-1β, IFN-γ, TNF-α) disrupt blood–brain barrier integrity, promote neuronal apoptosis, and drive glial proliferation (Hwang et al., 2006; Yang et al., 2017; Yuan et al., 2003), ultimately leading to macroscopic neuroanatomical damage. Notably, the excessive accumulation of inflammatory waste and associated neuropathology suggest impaired brain waste clearance (Lv et al., 2021). The glymphatic system, a recently characterized central neuron system waste clearance pathway, facilitates cerebrospinal fluid (CSF) influx, interstitial fluid (ISF)—CSF exchange, and metabolic waste removal via perivascular spaces (PVSs) (Iliff et al., 2012; Klostranec et al., 2021; Thomas et al., 2018). Our previous research utilizing diffusion tensor imaging along the perivascular space (DTI-ALPS) demonstrated abnormal perivascular ISF drainage in NPC patients following RT (Zheng et al., 2023). However, whether RT disrupts the critical glymphatic components of CSF influx and ISF-CSF exchange remains unexplored.

The choroid plexus (CP) plays a pivotal role in CSF homeostasis, blood-CSF barrier integrity, and neuro-immune communication (Mihaljevic et al., 2021; Solár et al., 2020). Located adjacent to the NPC radiation fields, its fenestrated epithelium makes it highly vulnerable to radiation-induced endothelial damage and cytokine-driven inflammation, which may disrupt CSF dynamics and exacerbating neurotoxicity (Calvo et al., 1987; Calvo et al., 1986; Solár et al., 2020). Animal studies indicate that radiation-induced CP hypertrophy correlates with BBB disruption (Bender et al., 2025). Clinically, CP enlargement has also been associated with neuroinflammation in multiple sclerosis (Kolahi et al., 2024) and Alzheimer’s disease (Jiang et al., 2024). Despite the anatomical proximity of the CP to NPC radiation fields, its role in RBI—specifically regarding volume changes and their relationship with WM integrity—remains uninvestigated in human cohorts.

Advances in diffusion magnetic resonance imaging, particularly free water imaging (FWI), have enable the quantification of the extracellular free-water fraction (FWF), a surrogate marker for neuroinflammation, vasogenic edema, and axonal degeneration (Finsterwalder et al., 2020; Pasternak et al., 2009; Shah et al., 2024; Wu et al., 2024). Elevated FWF reflects disrupted fluid homeostasis, as supported by its correlation with cytokine dysregulation in WM (Wu et al., 2024) and its association with vasogenic edema in multiple sclerosis and traumatic brain injury (Beaudoin et al., 2021; Butler et al., 2023; Gong et al., 2018). However, the spatiotemporal patterns of FWF alterations in WM tracts following RT in NPC patients remain undefined.

This study integrates CP volume and FWI to conduct a multi-dimensional assessment of glymphatic system dysfunction in NPC patients after RT. Our specific aims are to: (1) quantify CP volume changes in NPC patients after RT and their association with radiation doses of the temporal lobe and brainstem; (2) characterize FWF alterations within the critical WM tracts following RT; and (3) explore potential relationships between CP volumetric changes and WM FWF. We hypothesize that RT is associated with CP hypertrophy and FWF elevation in critical WM tracts, and that these changes correlate with radiation doses in NPC patients.

## 2 Materials and methods

### 2.1 Participants

This study comprised 101 patients with NPC, 45 in the before radiotherapy (pre-RT) group, and 56 in the post radiotherapy (post-RT) group. Tumors were staged utilizing the AJCC TNM classification, 7th edition (2009), ranging from T1N0M0 to T4N3M0 for both pre-RT and post-RT cohorts, with the latter also including T1N1M0.

The intensity modulated radiotherapy (IMRT) (Zhang et al., 2015) and conventional two-dimensional radiotherapy (2D-CRT) (Lai et al., 2011) were performed in all NPC patients. Refer to the established clinical guideline (Tang et al., 2021), all regions of interest, including tumor targets and organs at risk, were delineated on CT simulation images (slice thickness ≤3 mm) with patient immobilization using a thermoplastic mask. Target volumes were defined by integrating clinical and imaging findings, stratified by risk, and expanded with appropriate margins. Standardized anatomical definitions and multidisciplinary review ensured the accuracy and consistency of contouring for critical structures such as the brainstem and spinal cord. The detailed data of the RT regimen could refer to our previous works (Lin et al., 2017; Zhang et al., 2018).

For NPC patients classified as stages IIb to IVa-b, concurrent chemoradiotherapy, optionally supplemented with neoadjuvant or adjuvant chemotherapy, was recommended. This treatment strategy was administered 1–3 months before or after RT. The chemotherapeutic agents used may have included cisplatin, nedaplatin, paclitaxel, and fluorouracil, as detailed in Table 1 and Supplementary Table S1. This prospective study was approved by the Medical Research Ethics Committee of our Hospital and written informed consent was obtained from all subjects. The study was conducted in accordance with the Declaration of Helsinki (as revised in 2013).

**Table 1:** General clinical data and demographic date.

Clinical characteristic	Pre-RT group (*n* = 45)	Post-RT groups (*n* = 56)	*p* value
Age(years), mean ± SD	47.27 (9.51)	43.48 (10.17)	0.059
Gender, *n*			
Male	33 (73.33%)	44 (78.57%)	0.539
Female	12 (26.67%)	12 (21.43%)	
Clinical staging (UICC/AJCC2009)			
I/II	11 (24.44%)	12 (21.43%)	0.086
III/IV	34 (75.56%)	44 (78.57%)	
Teatment option			
Radiotherapy only, *n*	NA	5 (8.93%)	
Radio-chemotherapy, *n*	NA	51 (91.07%)	
Chemotherapy mode for patients treated with radio-chemotherapy			
Neoadjuvant and concomitant chemotherapy, *n*	NA	49 (96.08%)	
Others, *n*	NA	2 (3.92%)	
Chemotherapy regimens for patients treated with radio-chemotherapy			
PF/TP/TPF, *n*	NA	43 (84.31%)	
GP and others, *n*	NA	8 (15.69%)	

(1) NPC Nasopharyngeal carcinoma; TPF, docetaxel, cisplatin and fluorouracil; TP, docetaxel and cisplatin; PF, cisplatin and fluorouracil; GP, gemcitabine and cisplatin; NA, not available.

(2) Clinical stage were obtained according to the 7th edition of the UICC/AJCC (2009) TNM. Stage I: T1N0M0; Stage II: T0-1N1M0 and T2N0–1 M0; Stage III: T0-2N2M0 and T3N0-2 M0; Stage IV: T4N0-2 M0, or N3 or M1.

### 2.2 MRI acquisition and image assessment

MRI data collection was conducted prospectively utilizing a 3.0 T Siemens Magnetom Tim Trio scanner with a 32-channel head coil. The protocol included routine imaging sequences: Whole-brain T1-weighted images were acquired using a 3D MPRAGE sequence with the following parameters: 176 sagittal slices; slice thickness = 1.0 mm (no gap); matrix size = 256 × 256; field of view (FOV) = 256 × 256 mm; repetition time (TR) /echo time (TE) = 2300/2.98 ms; flip angle = 9°; isotropic voxel size = 1.0 mm^3^. DTI data were acquired using an echo-planar imaging sequence with the following parameters: matrix size = 128 × 128; FOV = 256 × 256 mm^2^; 85 axial slices with slice thickness = 2.0 mm and no gap (resulting isotropic voxel size = 2.0 × 2.0 × 2.0 mm^3^); TR/TE = 10,800/87 ms. Diffusion weighting was applied along 64 non-collinear directions with a b-value of 1,000 s/mm^2^, supplemented by one b = 0 s/mm^2^ volume.

### 2.3 Structural MRI processing

For structural image analysis, we employed the CAT12 toolbox (CAT12; http://dbm.neuro.uni-jena.de/cat) running through SPM12^1^ in MATLAB 2014a (MathWorks) to process all 3D T1 MRI datasets. The preprocessing pipeline included: (1) native-space segmentation of T1-weighted images into gray matter (GM), WM, and CSF compartments; (2) calculation of total intracranial volume (TIV) as the sum of GM, WM, and CSF volumes for each participant.

### 2.4 Choroid plexus volume evaluation

CP segmentation was conducted using a pre-trained U-Net model^2^ with T1-weighted images (Eisma et al., 2024), which have been validated in neurodegenerative disorders such as Parkinson’s disease (Pierobon Mays et al., 2024) and small vessel disease (Li et al., 2025). All T1-weighted images were non-linearly registered to the MNI 152 template using ANTs, with subsequent region-of-interest cropping around the CP guide by a probabilistic atlas. The model generated CP masks in standard space, from which bilateral and total CP volumes were calculated.

### 2.5 Free water fraction calculation

DTI images were preprocessed using FSL, including motion and eddy current correction, followed by brain extraction. Following established free water elimination approaches (Garyfallidis et al., 2014; Li et al., 2024; Pasternak et al., 2009), we employed DIPY package to implement a single-shell two-compartment model, generating both FWF maps (0–1 range, incorporating a fixed 37 °C water diffusion coefficient) and FW-corrected tensor maps. For tract-specific analysis, the images were affine-registered to JHU DTI template space (Hua et al., 2008) using FSL’s FLIRT. Mean FWF values were then systematically extracted for every WM tract defined in the template, enabling a comprehensive atlas-based evaluation across the entire WM skeleton.

### 2.6 Statistical analyses

Intergroup differences between pre-RT and post-RT groups were subsequently assessed using independent samples *t*-tests for age, TIV, CP volume, and FWF values, while gender was compared via chi-square tests. To delineate radiation-specific effects, general linear models (GLMs) were constructed with CP volume or FWF as dependent variables, adjusting for age, gender, and TIV as covariates. Tract-specific FWF group differences were FDR-corrected (*p* < 0.05) and tracts showing significant between-group differences were reported. Spearman correlation analysis was conducted to assess relationships between neuroimaging metrics (CP volume, FWF) and RT doses (maximum and mean doses to the temporal lobes and brainstem).

## 3 Results

### 3.1 Demographic characteristics

No significant differences were observed between the pre-RT and post-RT groups in terms of age (47.27 ± 9.51 vs. 43.48 ± 10.17 years, *p* = 0.059), gender (male: 73.33% vs. 78.57%, *p* = 0.539), and clinical staging (I/II: 24.44% vs. 21.43%, *p* = 0.086) (Table 1).

### 3.2 Choroid plexus volume analysis

**Figure 1:**
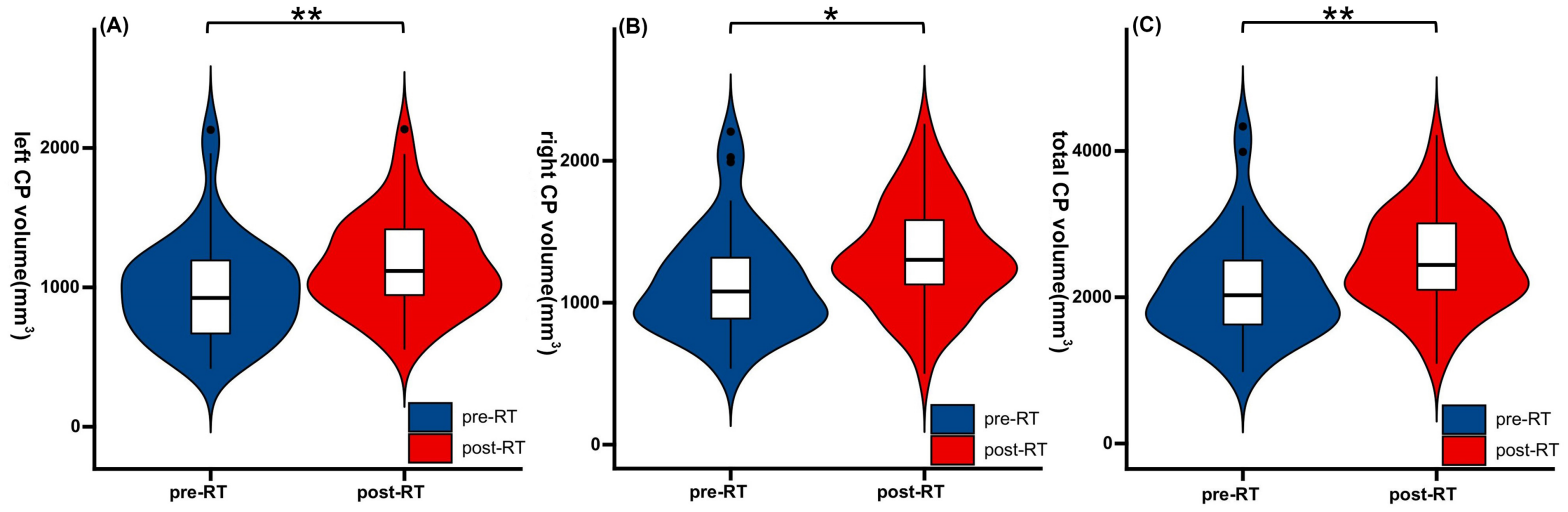
Choroid plexus volumetric changes in NPC patients after RT. Post-RT patients exhibited significant bilateral enlargement of choroid plexus (CP) volume compared to the pre-RT group. Panels illustrate total left CP volume **(A)**, right CP volume **(B)**, and total CP volume **(C)**. Values are presented as mean ± SD. CP, Choroid plexus; RT, Radiotherapy. **p < 0.01, *p < 0.05.

Compared to pre-RT group, the post-RT group exhibited a significant enlargement of bilateral CP (Table 2; Figure 1). Total CP volume increased by 18.2% (2165.57 ± 705.29 mm^3^ vs. 2560.56 ± 636.72 mm^3^, *p* = 0.006), with left and right CP volumes showing increases of 19.1% (1004.64 ± 362.68 mm^3^ vs. 1196.92 ± 334.53 mm^3^, *p* = 0.008) and 17.5% (1160.93 ± 377.89 mm^3^ vs. 1363.64 ± 365.84 mm^3^, *p* = 0.013), respectively.

**Table 2:** CP volume and FWF of between-group differences in NPC.

index	Pre-RT group (*n* = 45)	Post-RT group (*n* = 56)	*T*	*p* value
Total CP volume (mm^3^)	2165.57 (705.29)	2560.56 (636.72)	−2.802	0.006*
Left CP volume (mm^3^)	1004.64 (362.68)	1196.92 (334.53)	−2.711	0.008*
Right CP volume (mm^3^)	1160.93 (377.89)	1363.64 (365.84)	−2.537	0.013*
Middle cerebellar peduncle	0.511 (0.040)	0.542 (0.056)	−3.239	0.002*
Pontine crossing tract	0.417 (0.053)	0.454 (0.065)	−2.822	0.006*
Right corticospinal tract	0.539 (0.081)	0.594 (0.103)	−3.111	0.002*
Left corticospinal tract	0.570 (0.075)	0.631 (0.102)	−3.631	0.000*
Left medial lemniscus	0.458 (0.063)	0.500 (0.068)	−2.972	0.004*
Right inferior cerebellar peduncle	0.543 (0.087)	0.597 (0.106)	−2.896	0.005*

*FDR corrected with *p* < 0.05; CP, choroid plexus; FWF, free-water fraction; NPC, nasopharyngeal carcinoma; FDR, false discovery rate; RT, radiotherapy.

### 3.3 Free-water fraction alterations in white matter tracts

**Figure 2:**
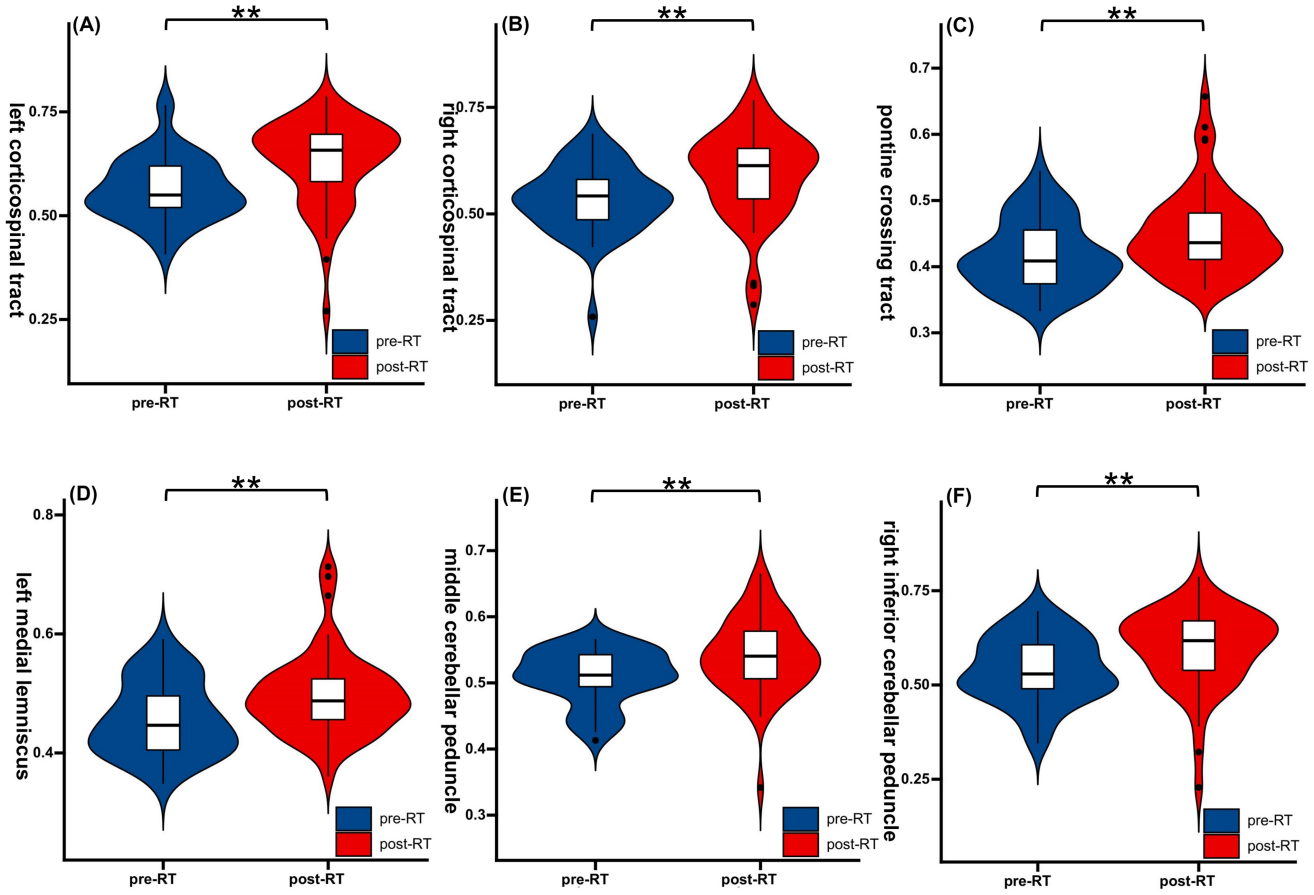
Radiation-induced alterations in FWF of white matter tracts. Post-RT patients demonstrated elevated FWF in critical white matter tracts, which include: left corticospinal tract **(A)**, right corticospinal tract **(B)**, pontine crossing tract **(C)**, left medial lemniscus **(D)**, middle cerebellar peduncle **(E)**, and right inferior cerebellar peduncle **(F)**. Color bars indicate FWF values (0–1 scale). Statistical significance: **p < 0.01 (FDR-corrected). FWF, free water fraction; RT, Radiotherapy.

**Figure 3:**
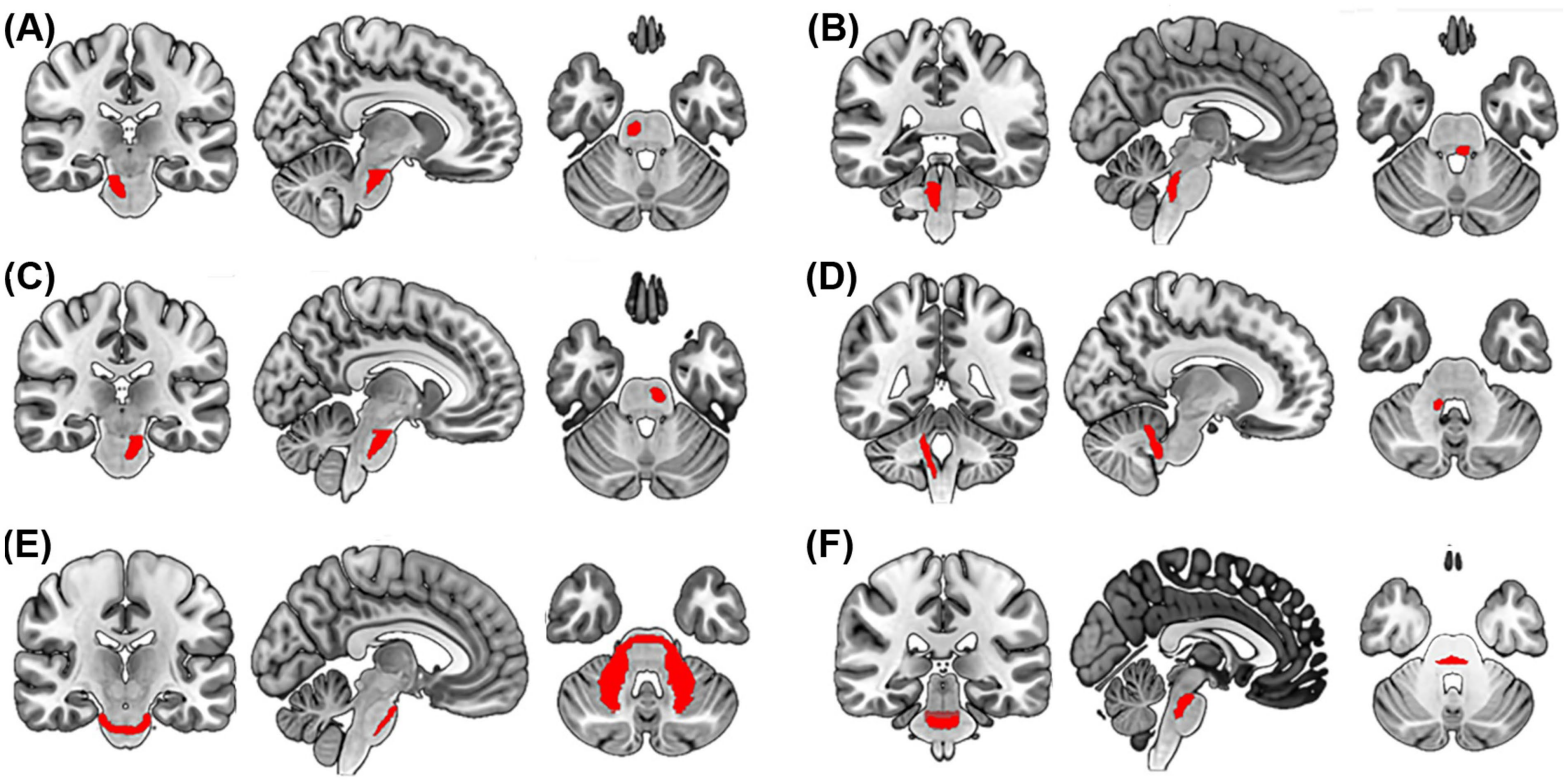
Spatial distribution of WM tracts with increased FWF in NPC patients after RT. Multiplanar views (coronal, sagittal, and axial) depict tracts exhibiting significant FWF elevation: left corticospinal tracts **(A)**, right corticospinal tracts **(B)**, right cerebellar peduncle **(C)**, pontine crossing tract **(D)**, middle cerebellar peduncle **(E)**, and left medial lemniscus **(F)**. Tracts are overlaid on the MNI152 brain template. WM, whiter matter; FWF, free water fraction; NPC, Nasopharyngeal Carcinoma.

A significant increase in FWF was observed across critical WM tracts in post-RT patients (Table 2; Figure 2). The most significant increases were observed in the pontine crossing tract (PCT) (ΔFWF = +8.9%, *p* = 0.006) and left corticospinal tract (CST) (ΔFWF = +10.7%, *p* < 0.001). Additional increases were observed in the right CST (ΔFWF = +10.2%, *p* = 0.002), middle cerebellar peduncle (MCP) (ΔFWF = +6.1%, *p* = 0.002), left medial lemniscus (ML) (ΔFWF = +9.2%, *p* = 0.004), and right inferior cerebellar peduncle (ICP) (ΔFWF = +9.9%, *p* = 0.005) (see Figure 3).

### 3.4 Correlation analysis

**Figure 4:**
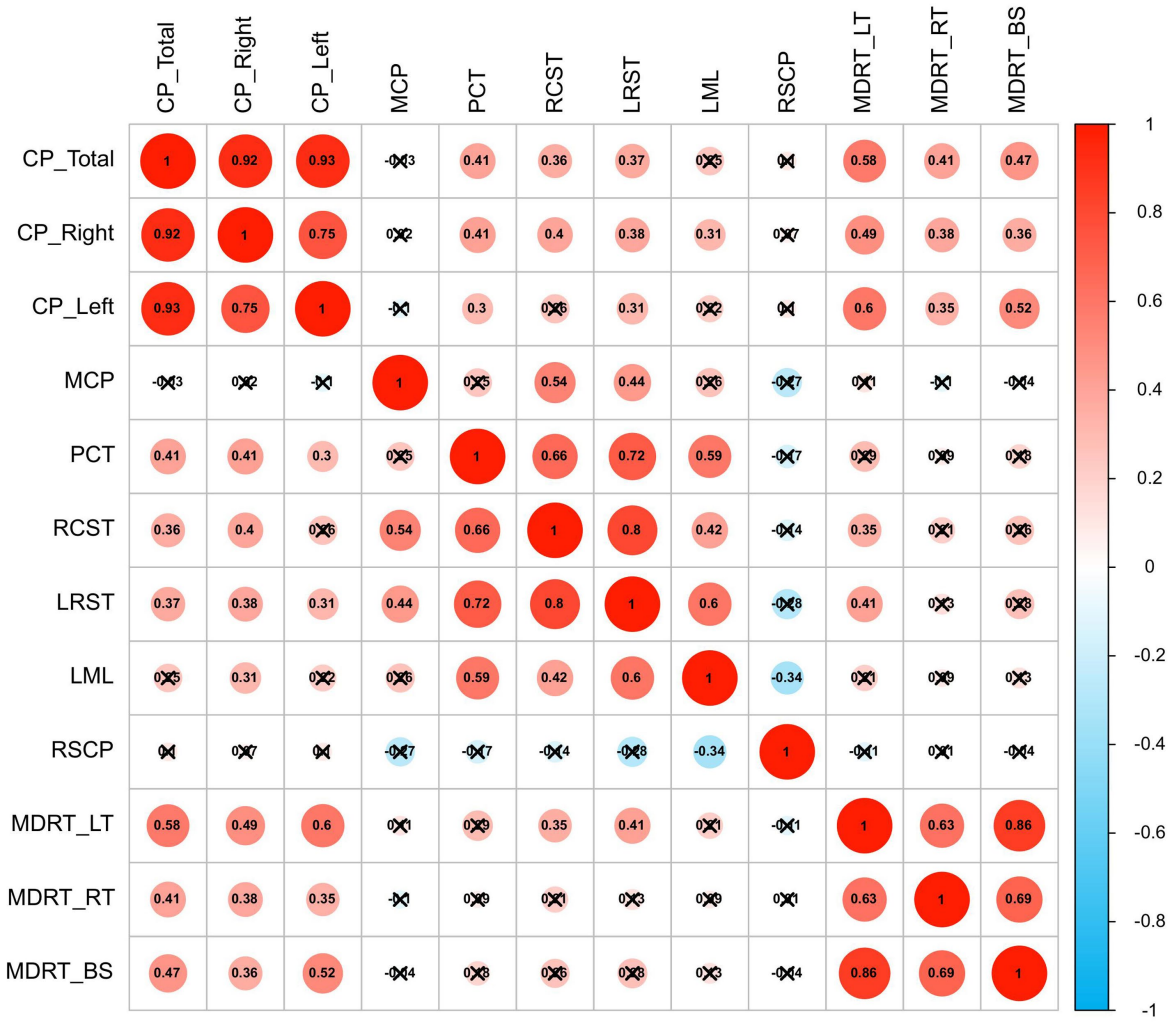
Heat map of the correlations between variables in post-RT group.

The radiation dose exhibited robust dose-dependent correlations with CP enlargement and elevated WM FWF (Figure 4). Brainstem maximum dose (MDRT_BS) significantly correlated with left CP volume (*r* = 0.516, *p*<0.001), right CP volume (*r* = 0.364, *p* = 0.014), and total CP volume (*r* = 0.466, *p* = 0.001). Similarly, right temporal lobe maximum dose (MDRT_RT) showed positive associations with left CP volume (*r* = 0.349, *p* = 0.019), right CP volume (*r* = 0.405, *p* = 0.006), and total CP volume (*r* = 0.382, *p* = 0.010). Notably, left temporal lobe maximum dose (MDRT_LT) demonstrated the positivity associations with left CP volume, right CP volume, and total CP volume (left: *r* = 0.599, *p* = 0.000; right: *r* = 0.585, *p* = 0.000; total: *r* = 0.490, *p* = 0.001). MDRT_LT also correlated significantly with elevated FWF in bilateral CST (left CST: *r* = 0.414, *p* = 0.005; right CST: *r* = 0.354, *p* = 0.017).

Furthermore, the mean FWF in the PCT and left CST correlated positively with left CP volume (PCT: *r* = 0.301, *p* = 0.044; left CST: *r* = 0.311, *p* = 0.038), right CP volume (PCT: *r* = 0.411, *p* = 0.005; left CST: *r* = 0.384, *p* = 0.009), and total CP volume (PCT: *r* = 0.405, *p* = 0.006; left CST: r = 0.374, *p* = 0.011). The FWF of right CST was positively correlated with total CP volume (*r* = 0.364, *p* = 0.014) and right CP volumes (*r* = 0.398, *p* = 0.007).

After adjusting for age, gender, and TIV, significant correlations remained: First, MDRT_BS and bilateral MDRT were correlated with bilateral and total CP volumes (*r* = 0.333–0.582; all *p* < 0.05); Second, MDRT_LT correlated with FWF in both the left and right CST (left: *r* = 0.381, *p* = 0.013; right: *r* = 0.313 *p* = 0.043). Finally, left CST FWF was correlated with bilateral and total CP volumes (left CP: *r* = 0.307, *p* = 0.048; right CP: *r* = 0.399, *p* = 0.009; total CP: *r* = 0.394, *p* = 0.010), and right CST FWF remained correlated with right CP volume (*r* = 0.342, *p* = 0.027).

Key findings include: MDRT_BS shows significant positive correlations: with left CP (*r* = 0.406, *p* = 0.006), right CP (r = 0.332, *p* = 0.026), and total CP volumes (*r* = 0.433, *p* = 0.003). MDRT_RT has positive associations: with left CP (*r* = 0.334, *p* = 0.025), right CP (*r* = 0.377, *p* = 0.011), and total CP volume (*r* = 0.382, *p* = 0.010). Notably, MDRT_LT demonstrates strong positive associations: with left CP (*r* = 0.570, *p* < 0.001), right CP (*r* = 0.477, *p* = 0.001), and total CP volume (*r* = 0.596, *p* < 0.01). It also correlates with elevated FWF in bilateral corticospinal tracts (left: *r* = 0.382, *p* = 0.012; right: *r* = 0.318, p = 0.038). The mean FWF of PCT positively correlates: with left (*r* = 0.326, *p* = 0.029) and total CP volume (*r* = 0.330, *p* = 0.027). The FWF of left CST has positive associations: with total (*r* = 0.350, *p* = 0.021) and right CP volumes (*r* = 0.353, *p* = 0.020). Color scale: red indicates positive correlation; blue indicates negative correlation. FDR - corrected for multiple comparisons. Abbreviations: MCP (pontine crossing tract); PCT (pontine crossing tract); LCST (left corticospinal tract); RCST (right corticospinal tract); LML (left medial lemniscus); RSCP (right superior cerebellar peduncle); MDRT_BS (brainstem maximum dose); MDRT_LT (left temporal lobe maximum dose); MDRT_RT (right temporal lobe maximum dose); CP (choroid plexus).

## 4 Discussion

Using multimodal magnetic resonance imaging, we investigated the changes in CP volume and FWF within WM tracts in NPC patients following RT. Our findings firstly demonstrate that post-RT NPC patients exhibit significant enlargement of the CP and increased FWF across multiple critical WM tracts, particularly in the PCT, left ML and bilateral CST. Correlation analysis revealed spatially distinct dose–response relationships between radiation exposure and these microstructural alterations. Specifically, MDRT_BS exhibited positive correlations with left CP, right CP volume, and total CP volume, while MDRT_LT was broadly associations with bilateral CP volume, total volume and the FWF of the left CST. Additionally, FWF in the right CST correlated positively with total CP volume and right CP volume.

Our findings show that post-RT NPC patients exhibit a significant increase in total CP volume as well as bilateral CP volume, consistent with our initial hypothesis. Compared with pre-RT NPC, the total CP volume increased by 18.2%, and the left and right CP were enlarged by 19.1 and 17.5% in post-RT NPC patients, respectively. This observation aligns with prior reports of CP volume enlargement approximately 9–11 months after RT for brain metastases (Reibelt et al., 2022). It is crucial to note that CP enlargement is a non-specific morphological alteration that may arise from multiple, potentially concurrent, pathophysiological processes (Jiang et al., 2024; Kolahi et al., 2024). These include reactive hyperplasia due to inflammation, fibrosis, angiogenesis, and dysregulation of CSF production and transport. Therefore, the observed volumetric increase should not be interpreted unidirectionally as a simple indicator of dysfunction.

We hypothesize that in the context of post-RT brain injury, CP enlargement may initially reflect a compensatory to radiation-induced neuroinflammation and blood-CSF barrier disruption, possibly through increased secretory activity to facilitate waste clearance (Balusu et al., 2016; Calvo et al., 1987). However, evidence from other neuroinflammatory and neurodegenerative conditions suggests that such a compensatory state may progressively transition into a maladaptive one over time (Choi et al., 2022; Solár et al., 2020). Chronic inflammation can induce fibrotic transformation, immune cell infiltration, and basement membrane thickening within the CP, ultimately impairing its permeability, reducing CSF secretion, and diminishing its neuroprotective and homeostatic functions (Marques et al., 2013; Prineas et al., 2016). Consequently, the core functional aspect hypothesized to be altered in our cohort is the CP’s integral role in CSF-ISF homeostasis—specifically, the dynamic equilibrium between CSF production, its influx into the parenchyma via perivascular spaces, and subsequent clearance of interstitial waste. Enlargement associated with fibrosis and inflammation could disrupt this equilibrium, leading to impaired CSF turnover and stagnation of extracellular fluid, as indirectly suggested by the correlation between CP volume and elevated FWF in white matter tracts. Definitive characterization of whether CP enlargement in this setting is predominantly adaptive or detrimental requires future longitudinal studies combining serial MRI with CSF biomarker analysis.

Another key finding is that the mean FWF within critical tracts (corticospinal tracts, cerebellar peduncles, brainstem fibers) was significantly increased in the post-RT NPC patients. FWF elevation serves as a sensitive imaging biomarker for underlying neuroinflammation, vasogenic edema, and axonal degeneration (Lin et al., 2021; Pasternak et al., 2009; Wu et al., 2024), confirming microstructural damage in these critical WM tracts. This aligns with established evidence that white matter injury is a hallmark of radiation-induced brain damage in NPC (Voon et al., 2021). Pathophysiologically, FWF elevation reflects dependent mechanisms of radiation injury (Lin et al., 2017), indicating an expansion of extracellular space linked to altered interstitial fluid dynamics (Pasternak et al., 2009). In the acute phase (days to weeks post-RT), microvascular damage (endothelial apoptosis, basement membrane thickening) disrupts the blood–brain barrier, permitting plasma extravasation that induces vasogenic edema. Progressing to the early-delayed phase (1–6 months), oligodendrocyte apoptosis triggers demyelination-reducing myelin-bound water retention-while concurrent axonal injury provokes axoplasmic edema, synergistically elevating FWF (particularly in radiation dose-dependent white matter tracts). In the late-delayed phase (>6 months), sustained microglial/astrocytic activation releases IL-6 and TNF-α, remodeling the extracellular matrix via glial scarring to perpetuate high FWF levels, establishing the substrate for neurocognitive impairment. This triad of mechanisms—vascular leakage initiating edema, demyelination weakening hydrostatic constraints, and chronic inflammation solidifying interstitial expansion—collectively mediates the dose-dependent WM injury reflected by increased extravascular water volume (Klistorner et al., 2022). Consequently, our findings underscore the clinical imperative for tractography-guided intensity-modulated RT to preserve eloquent pathways, warranting future longitudinal studies integrating diffusion tensor imaging with histopathological validation.

Furthermore, we observed a positive correlation between the post-RT enlargement of total CP volume and the increased FWF within the left CST. This association suggests that CP enlargement may impair normal CSF secretion and ISF dynamics, disrupting the critical CSF-ISF equilibrium. Mechanistically, altered CP function could affect AQP4-mediated CSF influx into the parenchyma, while FWF-reflective extracellular fluid stagnation may impede metabolic clearance. These findings indicate altered glymphatic function in post-RT NPC patients—a conclusion strengthened by our prior observation of a reduced DTI-ALPS index (Zheng et al., 2023), indicative of compromised perivenous drainage pathways. Collectively, these disruptions (impaired CSF influx, interstitial stagnation, and reduced drainage) may potentially contribute to *β*-amyloid accumulation and sustain neuroinflammation, providing a potential pathophysiological basis for the high incidence of cognitive decline in long-term NPC survivors. While this association highlights a plausible mechanism, establishing causality requires further validation through targeted animal models and molecular imaging.

Several limitations warrant consideration. First, the cross-sectional design precludes causal inferences; longitudinal studies following the same patients pre- and post-RT are needed to confirm temporal associations. Second, concurrent chemotherapy may confound radiation-specific effects, necessitating animal models and well-controlled cohort design to isolate RT-induced neurotoxicity. Third, the absence of cognitive assessments limits clinical correlation; future work should incorporate standardized neuropsychological testing (e.g., Montreal Cognitive Assessment, Wechsler Memory Scale) to explore relationships between CP/FWF changes and cognitive function. Fourth, the small sample size restricts subgroup analyses by disease stage or treatment modality. Five, due to the restricted field of view in nasopharyngeal CT scans, only the choroid plexus within the temporal lobes was visualized, limiting whole-brain assessment. Future studies should prospectively delineate the choroid plexus using whole-brain MRI to enable complete dose–volume analysis. Finally, the lack of histopathological validation necessitates cautious interpretation of FWF changes.

## 5 Conclusion

This study identifies CP enlargement and widespread elevation of WM FWF as distinct hallmarks of radiation-associated brain injury in NPC patients, exhibiting robust dose–response relationships. Critically, the observed interplay between CP enlargement and WM FWF elevation points towards glymphatic dysfunction as a novel and integrative mechanism potentially underpinning post-RT RBI.

## Data Availability

The original contributions presented in the study are included in the article/Supplementary material, further inquiries can be directed to the corresponding authors.

## Footnotes

1
https://www.fil.ion.ucl.ac.uk/spm/software/spm12/

2
https://github.com/hettk/chp_seg
